# Efficient push-grasping for multiple target objects in clutter environments

**DOI:** 10.3389/fnbot.2023.1188468

**Published:** 2023-05-12

**Authors:** Liangdong Wu, Yurou Chen, Zhengwei Li, Zhiyong Liu

**Affiliations:** ^1^School of Artificial Intelligence, University of Chinese Academy of Sciences, Beijing, China; ^2^Institute of Automation, Chinese Academy of Sciences, Beijing, China; ^3^Cloud Computing Center, Chinese Academy of Sciences, Dongguan, Guangdong, China

**Keywords:** Deep Learning in Robot Manipulation, reinforcement learning, intelligent system, push-grasping, robot control

## Abstract

Intelligent manipulation of robots in an unstructured environment is an important application field of artificial intelligence, which means that robots must have the ability of autonomous cognition and decision-making. A typical example of this type of environment is a cluttered scene where objects are stacked and close together. In clutter, the target(s) may be one or more, and efficiently completing the target(s) grasping task is challenging. In this study, an efficient push-grasping method based on reinforcement learning is proposed for multiple target objects in clutter. The key point of this method is to consider the states of all the targets so that the pushing action can expand the grasping space of all targets as much as possible to achieve the minimum total number of pushing and grasping actions and then improve the efficiency of the whole system. At this point, we adopted the mask fusion of multiple targets, clearly defined the concept of graspable probability, and provided the reward mechanism of multi-target push-grasping. Experiments were conducted in both the simulation and real systems. The experimental results indicated that, compared with other methods, the proposed method performed better for multiple target objects and a single target in clutter. It is worth noting that our policy was only trained under simulation, which was then transferred to the real system without retraining or fine-tuning.

## 1. Introduction

Robotic grasping and manipulation in an unstructured, clutter-filled environment is a challenging research subject. In such a scenario, the target tends to be tightly wrapped around the non-target objects. Thus, it is hard to execute effective grasping. To get feasible operation space for grasping, the system must first focus on the cluttered scene and then separate the target from other objects. Therefore, the current common methods (Boularias et al., 2015; Bauza and Rodriguez, 2017) include pushing as a pre-grasping operation. Although pushing and grasping are two different manipulative skills, the synergy of grasping and pushing actions remains challenging. Synergizing these two actions will improve the robot's ability to efficiently perform robotic manipulation in the midst of clutter.

There have been some studies on how to learn cooperative manipulation policies to achieve robotic push-grasping. Zeng et al. (2018) used the parallel training architecture of the grasping network and pushed the network to learn the push-grasping policy. Dogar and Srinivasa (2010), Hang et al. (2019), and Song and Boularias (2020) devoted themselves to pre-grasping operations to assist in grasping tasks. Deng et al. (2019) designed a grasping evaluation mechanism, but they lacked target orientation and preferred push-grasping tasks with an unknown target. According to Yang et al. (2020), the mask of the target object, which to a certain extent realizes the coordinated operation of pushing and grasping for a target in clutter, was used as the network input.

A target-oriented grasping task, that is, grasping the target object from the cluttered scene, needs to integrate the information of the target object into the pushing-grasping collaborative policy, so it is more challenging. In the study by Kiatos and Malassiotis (2019), a pushing policy was obtained using Q-learning to assist in the realization of target grasping. The motion evaluation mechanism and classification mechanism were designed by Yang et al. (2020) for motion primitives to achieve pushing and grasping around the target object. To improve the training efficiency and system performance, a three-stage training method of alternate grasping, pushing, and push-grasping was proposed by Xu et al. (2021). In the study by Huang et al. (2021b), combining a prediction network and Monto-Caro tree search, a model-based learning method was used to predict the shortest push sequence required to grasp the target object. All the above studies were conducted around the push-grasping task of the single target object in clutter. Multi-target grasping in clutter scenes was also common in real life. An intuitive way is to approach each target using the single target method one by one, but the efficiency of the actions may not be optimal. At the beginning of the study, we believed that there should be an optimal choice of actions, that is, to create the grasping space successively or simultaneously for all targets with the least amount of pushing actions. To achieve this, we needed to take into account all the targets, including their states, rewards, and robot actions, and then determine a feasible approach. This study focused on a multi-target push-grasping task in a clutter scenario, treated the task as a self-supervised reinforcement learning problem, and proposed a method based on policy learning for efficient multi-target synergy of push and grasp. Our approach was analogous to that of Zeng et al. (2018) and Xu et al. (2021). However, the experimental results showed that the direct application of these state-of-the-art methods makes it difficult to obtain satisfactory results for multi-target push-grasping. Hence, we improved and realized the extension of push-grasping synergy to multi-target tasks in clutter scenarios. To be specific, we performed the following steps: first, the image needed to be segmented and fused to obtain the multi-target mask, and the multi-target mask, RGB, and depth images were used as the network input. Second, we defined the graspable probability (GP) for the target object. This definition was evaluated according to the maximum output of the grasping network. The larger the value, the more likely the target was to be grasped. Finally, we set a reward mechanism based on the GP. In concrete terms, if the GP of a target object was increased after pushing or grasping, it was considered that the previous action was beneficial for the target object.

Moreover, if other target objects met this requirement, additional rewards were given during the training, which is different from the single-target policy learning. Therefore, we incorporated the multi-target states into the training process so that the policy could gradually master the efficient pushing and grasping of the targets. We conducted experiments in both simulations and the real system, and the experimental results indicated that, compared with other methods, the proposed method has better performance not only for multiple target objects but also for a single target in clutter; therefore, our system has a comprehensive capability and generalization from sim-to-real. To the best of our knowledge, in the field of policy learning, existing studies may not include multi-target push-grasping in clutter. In summary, the main contributions of this study are the following:

(1) We fused the masks of each target and defined the graspable probability under multi-target, as well as a reward mechanism that provides a training paradigm for a multi-target push-grasping synergy policy.

(2) The proposed method could accomplish the push-grasping task for both single and multiple target(s) in clutter.

(3) We evaluated the performance of the learned system in clutter scenarios not only for simulation but also for the real world. Experimental results indicated that the proposed method is more effective.

## 2. Related work

### 2.1. Grasping

Robotic grasping methods can be divided into analytical and data-driven categories (Bohg et al., 2013). The analytical method relied more on precise physical configuration and a detailed mechanical model to obtain the grasping feasibility prediction based on the force-closed type (Rodriguez et al., 2012). However, due to the difficulty associated with accurately obtaining physical properties (friction, etc.) and ensuring the accuracy of 3D object model construction, more researchers are gradually turning to data-driven methods to achieve direct mapping from vision to motion (Mahler et al., 2017; Choi et al., 2018). Meanwhile, sensor research based on deep learning has been carried out (Ovur et al., 2021; Qi et al., 2021, 2022). More data-driven approaches focused on a single object in a scattered scenario (Mahler and Goldberg, 2017; Kalashnikov et al., 2018; Lu et al., 2020; Sarantopoulos et al., 2020; Zhang et al., 2023). Researchers have recently started to apply this method to object grasping in clutter scenes (Boularias et al., 2014; Ten Pas and Platt, 2018). Fang et al. (2020) introduced a large-scale training standard for general object grasping. Moll et al. (2017) presented a grasping motion planning method. Ten Pas and Platt (2018) trained CNN to detect 6D grasping posture in the point cloud. However, these methods only use grasping without other types of action; therefore, their ability to deal with complex and chaotic environments is limited. Therefore, the combination of grasping pre-operation (such as pushing) and grasp may be better for robot grasping tasks in clutter scenarios.

### 2.2. Push-grasping

For non-prehensile manipulation, Cosgun et al. (2011) from a model-driven perspective and Danielczuk et al. (2018) from a data-driven perspective aimed to reduce the impact of uncertainties such as collisions. The combination of pushing and grasping further improved the robot's operating system (Boularias et al., 2015) to rearrange the disordered objects by pushing for subsequent grasping (Deng et al., 2019; Huang et al., 2021a). A representative study is that of Zeng et al. (2018), in which researchers proposed a model-free deep reinforcement learning framework to realize push-grasping synergy through parallel network architecture. Deng et al. (2019) evaluated whether the target is suitable for grasping. However, if grasping is unsuitable, a push was performed to obtain a better grasping space. Huang et al. (2021a) trained the neural network to predict the action and state after the execution of the push to distinguish whether grasping should be performed.

The above methods focused on the push-grasping task with an unknown target object. Comparatively, there are few studies on the push-grasping synergy for targets in the cluttered scene, except for Kiatos and Malassiotis (2019), Kurenkov et al. (2020), Yang et al. (2020), Xu et al. (2021), and Huang et al. (2021b). Kiatos and Malassiotis (2019) used Q-learning to train a pushing policy to achieve the separation of target objects in clutter. Kurenkov et al. (2020) provided rewards according to occlusion changes to train the pushing policy. Based on Baye's strategy to promote the exploration of invisible targets to expose them to the visual field, Yang et al. (2020) combined the action evaluation mechanism and the classification mechanism to achieve pushing and grasping around the target object. Xu et al. (2021) used the idea of HER (Andrychowicz et al., 2017) to relabel the grasping condition that meets the requirements to improve training efficiency. A three-stage training method was adopted to improve the performance of the push-grasping system. Combined with a state prediction network, Huang et al. (2021b), used a model-based learning method, based on the Monto-Caro tree search, to realize that the learned policy can predict the shortest push sequence required to grasp the target.

All of the above target-oriented push-grasping methods were aimed toward a single target. It is common in real life to find multiple target objects in clutter scenarios. The repeated application of a single target push-grasping method may not be the optimal choice for the overall manipulation of pushing and grasping. Our approach was data-driven, similar to the above methods, and the training architecture was similar to that of Zeng et al. (2018), both of which are parallel networks of pushing and grasping. However, we added the fusion of target masks to solve the multi-target recognition problem. Moreover, the definition of graspable probability was provided to better guide policy learning. To enable the policy to comprehensively consider each target state, the reward mechanism was set up based on the graspable probability. Thus, the learning paradigm and the specific method for multi-target push-grasping synergy were established.

## 3. Preliminaries

### 3.1. Problem formulation

To address the problem of multi-object push-grasping in clutter scenarios, the following definition has been provided:

*Definition 1*. A multi-target push-grasping task in clutter scenarios means that the robot takes several objects as retrieval targets from a series of densely disordered objects and obtains each target one by one with the least number of actions through limited pushing and grasping.

Objects in a cluttered scene, including multiple targets, may have different shapes, sizes, colors, weights, and other physical properties. For the setup of the experimental platform, we made the following basic assumptions: (1) the hardware included a UR3 robot equipped with a robotic two-finger 85 parallel gripper at the end, a flat workspace, and a Kinect camera above the workspace; (2) the objects were rigid and could adapt to the gripper's operation, that is, a straight-line pushing action and a top-down grasping action; (3) the working space was a fixed space within the field of view of the camera. If an object fell out of the working space, it could no longer be recognized and operated upon.

### 3.2. Primitive actions

Similar to previous studies (Zeng et al., 2018; Xu et al., 2021), we used the primitive actions of pushing and grasping instead of continuous action exploration. *a*_*M*_*g*__(*x, y*, θ) represents the grasping action primitively performed at a pixel (*x, y*) and θ represents the rotation angle of the gripper along the z-axis. (*x, y*) can be any pixel under the scene 224 × 224 of the workspace plane, and the center position of the gripper needs to be moved to this point.θ represents one of the 16 evenly divided portions 0~2π. When the gripper reaches the corresponding position, it moves down to the grasping position, executes the closure of the gripper, and then moves the target object outside the working space.

In the clutter scenarios, the target is often tightly wrapped by other objects; therefore, it is difficult to grasp directly. At present, the push action should be performed first to create grasping space. For the push action *a*_*M*_*p*__(*x*_0_, *y*_0_, *x*_1_, *y*_1_), the robot terminal gripper will move horizontally and straight from the initial point (*x*_0_, *y*_0_) to (*x*_1_, *y*_1_) to the point of closure.

## 4. Methods

**Figure 1:**
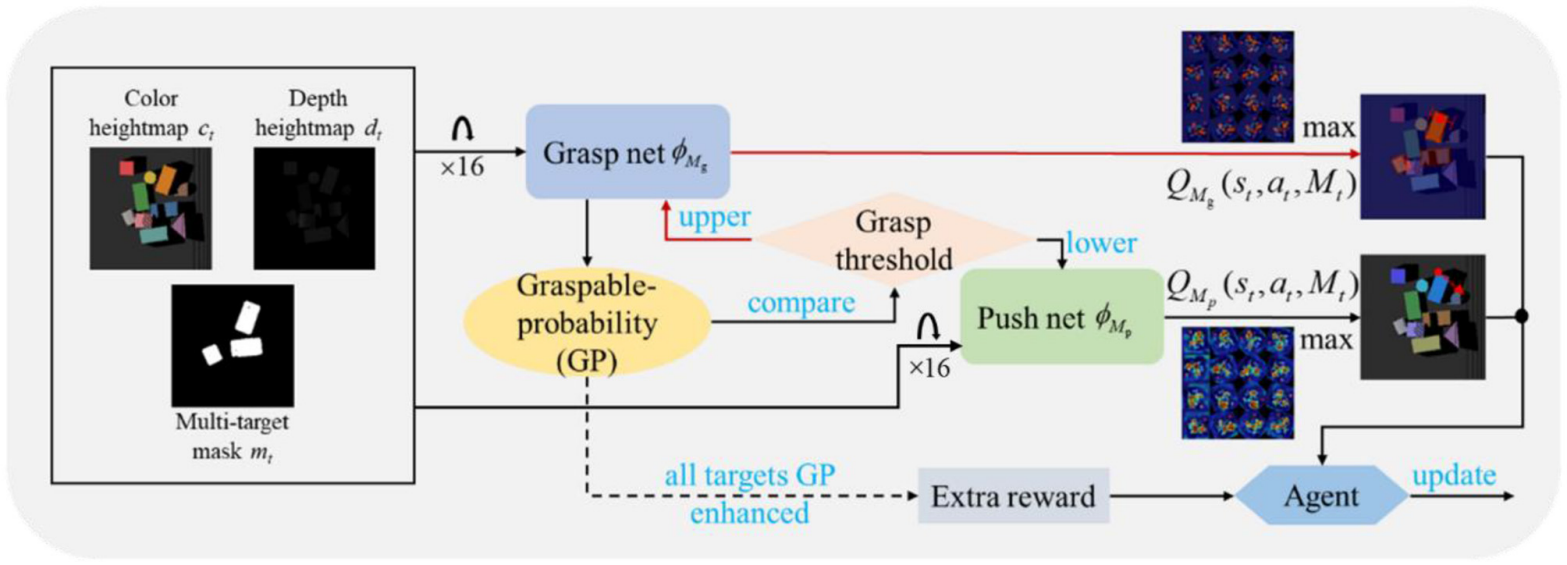
Overview of the whole system. We fused the mask of each target, provided a reasonable definition of graspable probability (GP), and considered the impact of each target in the reward function to realize the efficient multi-target push-grasping synergy (MTPG). When the system started running, the camera captured the RGB-D image of the clutter scenario. The input from the networks included three types of image information, as shown in the figure. The pixel-wise grasp Q maps that grasp network outputs were used as the quantized value of GP. If the GP of a certain target is greater than grasp threshold, a grasp action will be performed against the target; if the GP of multiple targets exceeds the threshold simultaneously, the action corresponding to the maximum grasp Q maps will be performed; otherwise, a push action will be performed. During the execution of the task, if the GP of all targets is enhanced, and the agent will receive an extra reward.

Objects in cluttered scenes were often tightly packed together. When the robot wanted to grasp, it first had to execute the pre-operation (pushing, etc.) to create the grasping space. If there was more than one target in these objects, the synergy of pushing and grasping was performed around each target. Before a certain target was successfully captured, any action the robot performed was likely to affect the subsequent operations of other targets, leading to a larger total number of actions for all targets to be captured. We were interested in ways to minimize the total number of actions so that the manipulation was as efficient as possible. To address this challenge, we proposed a model-free, self-supervised reinforcement learning method that trains neural networks to obtain a cooperative push-grasping policy. An overview of our system is shown in Figure 1.

We modeled the multi-target push-grasping problem as a Markov decision process and added a new symbol*M*_*t*_to represent the multi-target states, including color heightmap *c*_*t*_, depth heightmap *d*_*t*_, and multi-target mask *m*_*t*_. In this form, we defined the policy, reward, and Q-function as π(*s*_*t*_|*M*_*t*_), *R*(*s*_*t*_, *a*_*t*_, *M*_*t*_) and *Q*_π_(*s*_*t*_, *a*_*t*_, *M*_*t*_), respectively.

We adopted the parallel network architectures of pushing and grasping to realize the synergy, where ϕ_*M*_*g*__ represents grasp network and ϕ_*M*_*p*__ represents push network. The input of the network was the rotated heightmaps in the form of a total of 16 rotations, with each rotation of 22.5° corresponding to 16 different grasping angles based on the z-axis and 16 different horizontal pushing directions. The output was 32 Q-values of pixels (16 Q-values from ϕ_*M*_*g*__, others from ϕ_*M*_*p*__), and each Q-value represented the expected future reward of the corresponding primitive action if it were executed. The execution of the grasp action was determined by the maximum Q value output from the grasp network ϕ_*M*_*g*__, and the same logic as the push action by the push network ϕ_*M*_*p*__ was applied.

Multi-target grasp was the ultimate goal of the task, but the purpose of the pushing action was to create grasping space. Therefore, we defined the graspable probability (GP) of the target and the grasp threshold to measure the effectiveness of executed actions. As the training proceeded, the threshold tended to converge. When the push action was executed once or more, and if the GP of one target exceeded the threshold, the system executed the grasp action. Then, the system continued pushing GP to exceed the threshold of other targets.

The efficiency of the synergy policy was reflected in the implementation of only *n* grasp actions for *n* targets to complete the task with as few push actions as possible. We made some specific adjustments to the reward function for policy learning. In short, except for the sparse reward after each action execution, we gave additional rewards according to whether the GP of each target at the current time had been enhanced compared to the previous time.

### 4.1. Multi-mask

**Figure 2:**
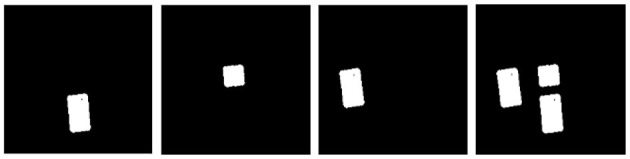
Mask fusion of multiple targets.

To implement multi-target push-grasping, each target needed to be effectively recognized first. After the RGB-D images of the scene were obtained by the camera, the target information could not be known for the first time; therefore, image segmentation was required. In the studies by Xu et al. (2021) and Huang et al. (2021a), a mask segmentation method was used to obtain the image information of a single object. However, for our research problem, the parallel training of multiple single targets greatly increased the processing load of the GPU. Therefore, we organically integrated all the target masks and reconstructed the multi-mask image information, including all targets. This is shown in Figure 2.

### 4.2. Graspable probability

For multi-target push-grasping in clutter scenes, the optimal situation was that one grasp was executed for one target; in other words, the total number of actions was *n* for *n* targets to complete the task. However, in direct grasping, it was hard to capture the target due to the tight wrapping of non-target objects. Therefore, we defined a metric to measure whether the target can be grasped, the graspable probability (GP):*G*_*p*_(*i*_*t*_), where *i* was one of any targets.

GP can be quantified by the Q value, which is output by the grasp network. The larger the Q value is, the greater the GP is. The reason that we normalized it was to dig out the actual meaning of the grasp Q value. Specifically, if the maximum Q-map value of the grasping network ϕ_*M*_*g*__ was less than the grasp threshold, the pushing action was performed to create more grasping space to improve the GP; otherwise, the grasping action was performed.

The purpose of multi-target push-grasping is to increase GP of every target through push action, rather than only for a certain target, instead just starting from the first target whose GP is more than grasp threshold to perform grasp action. After successful grasp, the system then follows the same logic to continue to push and grasp, until all targets are grasped successfully.

### 4.3. Reward for multi-target

To make the policy learn to comprehensively consider the push-grasping action for each target in the training process, reduce the impact of the execution of each action on the state of subsequent targets, and achieve the multi-target grasp with the shortest number of actions, we set up an additional reward. Specifically, if the graspable probability of each target at the current moment was higher than the term at the previous moment, an extra reward *R*_*M*_*e*__was provided as follows:


(1) $$R_{M_{e}}=\{\begin{array}{l} 0.5,\begin{array}{ll} \; & \frac{1}{n}\sum_{i=1}^{n}G_{p}(I_{t+1})>G_{p}(I_{t}),I\in \{one\;of\;n\;targets\} \end{array}\\ 0,\begin{array}{ll} \; & otherwise \end{array} \end{array}$$

### 4.4. Multi-target push-grasping synergy policy training

*Grasp training*. At the initial stage, we only trained the grasp network. *n* objects were randomly dropped in the workspace, where the number of target objects was *a* (< *n*). We took the form of sparse rewards:


(2) $$R_{M_{\text{g}}}=\{\begin{array}{l} 1,\begin{array}{ll} \; & grasp\;a\;certain\;target\;successfully \end{array}\\ 0,\begin{array}{ll} \; & otherwise \end{array} \end{array}$$

In addition, to enhance the training efficiency, we relabeled the wrong grasping experience (grasping non-target objects) and saved it in the replay buffer for training. The Q value of the grasp network tended to converge, which was regarded as the grasp threshold $Q_{g}^{*}$. In the subsequent training, *G*_*p*_(*i*_*t*_) had to be greater than this threshold before we executed the grasp action.

*Pushing and grasping coupling training*. The key point is to have as few push movements as possible to achieve an efficient push and grasp. The agent performed the push action when *G*_*p*_(*i*_*t*_) of each target did not exceed the grasp threshold $Q_{g}^{*}$. The pushing reward function took the following form:


(3) $$R_{M_{p}}=\{\begin{array}{l} 0.5,\begin{array}{ll} \; & change\;states\;of\;targets \end{array}\\ 0,\begin{array}{ll} \; & otherwise \end{array} \end{array}$$

Changing states indicate that the orientation or position of a certain target object has changed. However, to improve the GP of each target, it was necessary to motivate the agent further to make a more efficient motion decision; thus, the multi-target reward mentioned in Formulation (1) was added.

To improve the cooperativity of the push-grasp policy, we conducted pushing and grasping coupling training. At this stage, the grasp threshold remained unchanged, and the two networks were trained following the previous reward function and multi-target reward. The parameters of the grasping network were trained based on the previous stage. This training process could gradually optimize the performance of the push-grasping network. The environment reset of the training episode was consistent with the grasp training.

## 5. Experiments

In this section, we conducted a series of experiments to evaluate our system. The objectives of the experiments were as follows: (1) to verify that our policy can effectively realize multi-target push-grasping in clutter scenes, (2) to indicate whether our approach is still effective for single-target conditions, and (3) to investigate whether our system can be generalized to real-world experiments. The video is available at https://www.youtube.com/watch?v=Cp71V-29mgs.

### 5.1. Implementation details and simulation setup

Our network included three parallel networks with the same structure, consisting of a fully convolutional network (FCN) including 121-layer DenseNets (Huang et al., 2017) and ImageNet (Deng et al., 2009), which was used for feature extraction from input (color heightmaps, depth heightmaps, goal masks), and outputs each pixel's Q value. FCN uses batch normalization (Ioffe and Szegedy, 2015), is bilinearly upsampled, and includes two 1 × 1 convolutional layers with ReLU (Nair and Hinton, 2010). The loss function was set up the same as VPG (Zeng et al., 2018).

The Adam optimizer was utilized for network training, with a fixed learning rate10^-4^, a momentum of 0.9, and weight decay2^-5^. Our future discount γ was a constant 0.5. For the exploration strategy, we used ε-greedyand ε initialized at 0.5, then annealed it to 0.1.

**Figure 3:**
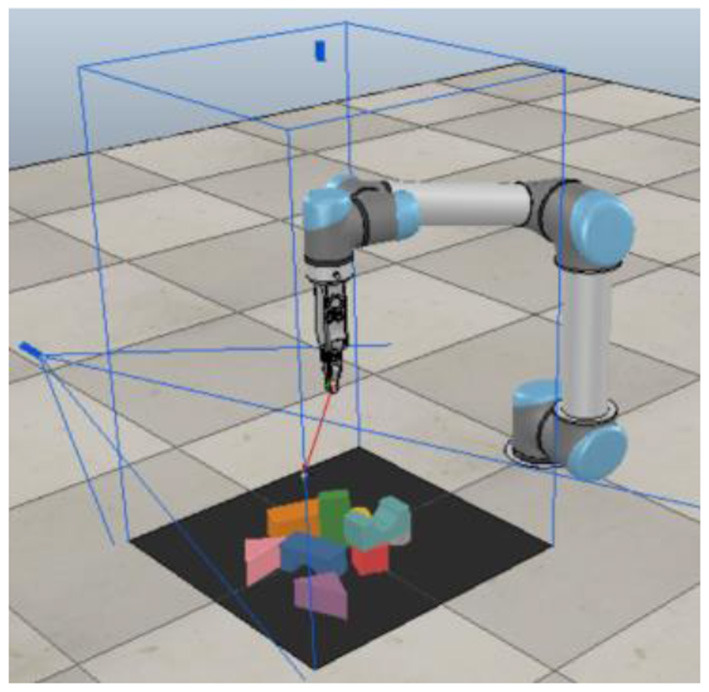
Multi-target push-grasping synergy policy learned in the V-REP simulation environment by the UR5 robot.

The simulation experiment used a UR5 robot equipped with an RG2 gripper, as shown in Figure 3. The simulation software is V-REP, with Bullet Physics 2.83 for dynamics configuration and using V-REP's built-in inverse dynamics module for robot action planning.

### 5.2. Baseline methods and evaluation metrics

We compared the proposed method with the following baseline approaches.

Grasp only is a method that uses grasp only without pushing action and uses an FCN network to train a greedy, deterministic grasping policy.

Goal-Conditioned VPG (gc-VPG) is an expanded version of VPG (Zeng et al., 2018), incorporating target masks to train policy. In this approach, two parallel DQN network architectures are used to predict pushing and grasping, respectively, and the corresponding action with the highest Q value is executed.

Goal-Oriented push-grasping (GOPG) (Xu et al., 2021). This method is modified on the basis of VPG, and the policy training is divided into three stages. The relabeling borrowed from HER (Andrychowicz et al., 2017) is used to improve training efficiency.

Baseline methods were originally proposed for single-target pushing and grasping in clutter scenarios. Hence, the environment is still a single target when we use it for policy training. In the test for multi-target pushing and grasping, baselines sequentially captured each target one by one.

#### 5.2.1. The ablation studies

To explore whether VPG and GOPG also have the potential for multi-target pushing and grasping, we made some adjustments to their method framework, changed the single-target mask to the multi-target mask, and retrained the policy to conduct compared experiments, that is, gc-VPG with multi masks (gc-VPG-mm) and GOPG with multi-masks (GOPG-mm).

For the test, we used the following four evaluation metrics to measure the system.

#### 5.2.2. Completion

The percentage of completed tests over total tests. For the multi-target push-grasping task, we stipulated that each target should not be executed for more than 10 times of invalid push (after an invalid push, the state of each target does not change) or failed to grasp 10 times before capture. Otherwise, it was deemed incomplete. When all targets were retrieved, the mission is considered complete.

#### 5.2.3. Grasp success rate

The ratio of the number of successful grasps over the total number of grasps.

#### 5.2.4. Motion number

The total number of pushing and grasping actions executed to obtain targets.

#### 5.2.5. Action efficiency

The ratio of the number of targets to the number of actions before completion.

Among these four metrics, the lower the motion number, the better. Others are higher and better.

### 5.3. Simulation experiments

**Figure 4:**
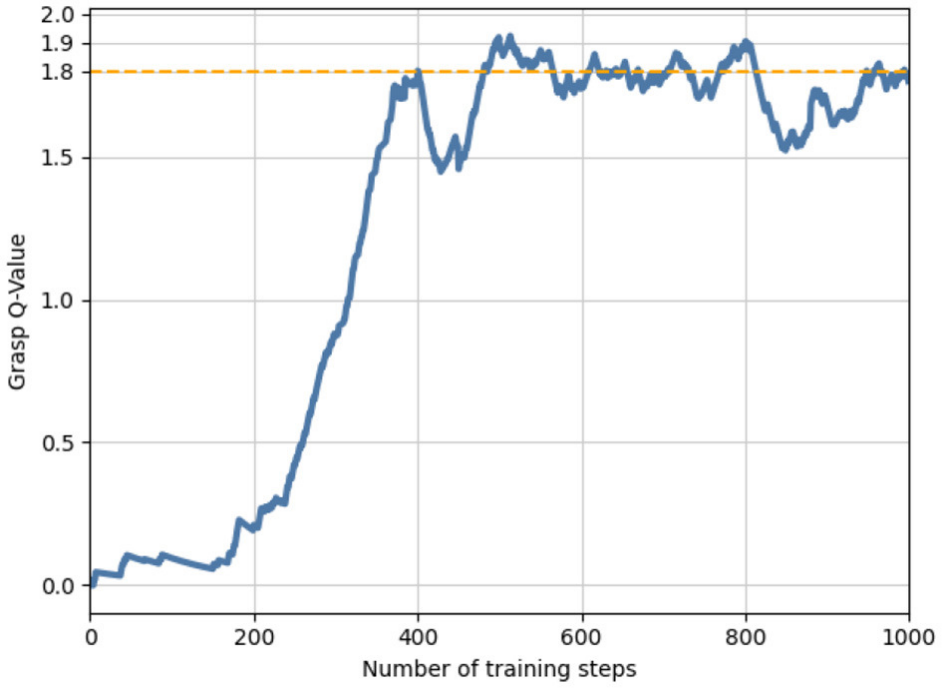
A preliminary grasping network and grasping threshold were obtained by grasping training in the initial stage.

For grasp training, 10 objects were dropped randomly into the workspace, and three of them were targets for 1,000 episodes. After approximately 500 steps of training, the grasping Q value could be stable at 1.8; therefore, we considered it the grasping threshold $Q_{g}^{*}\text{=}1.8$, as shown in Figure 4. In the subsequent pushing and grasping coupling training, the threshold was used to determine whether the action was pushing or grasping; that is, a grasp action was performed when the prediction of the Q value was greater than the threshold. Otherwise, a push-action operation was performed.

**Figure 5:**
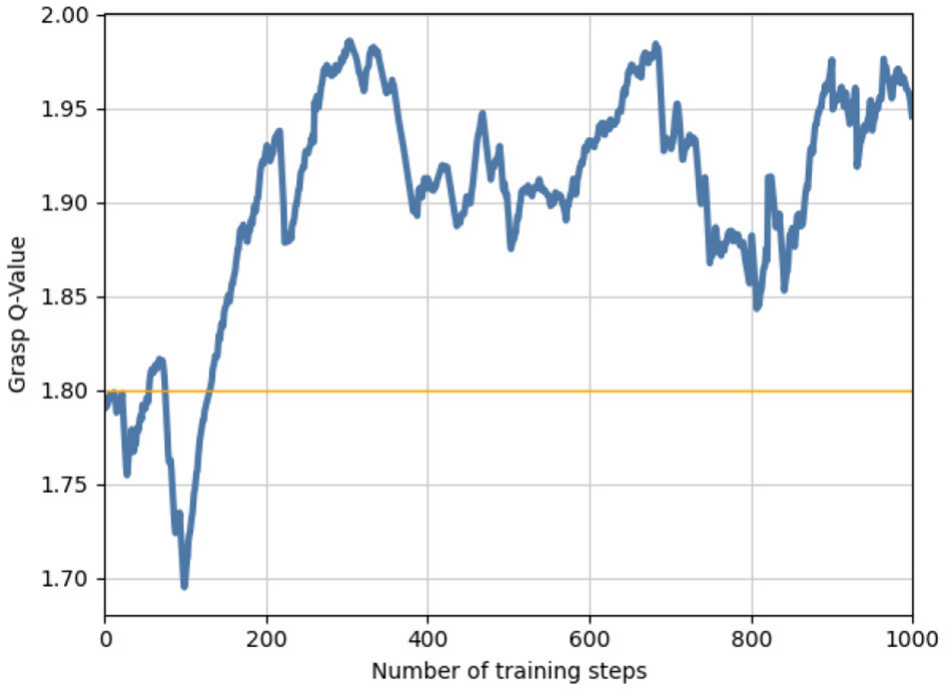
During coupling training, we ran 1,000 steps, and the policy gradually acquired the ability to grasp multiple targets with a probability greater than the threshold.

For push-and-grasp coupling training, we set up an additional reward mechanism to enable the policy to gradually master the discrimination of multi-target states and thus give the optimal decision in terms of overall action efficiency. As the training went on, after executing actions, the graspable probability of all targets was gradually improved, and the extra rewards also slowly increased, which also indicated that the policy's ability to multi-target push-grasping was improved, as shown in Figure 5.

**Figure 6:**
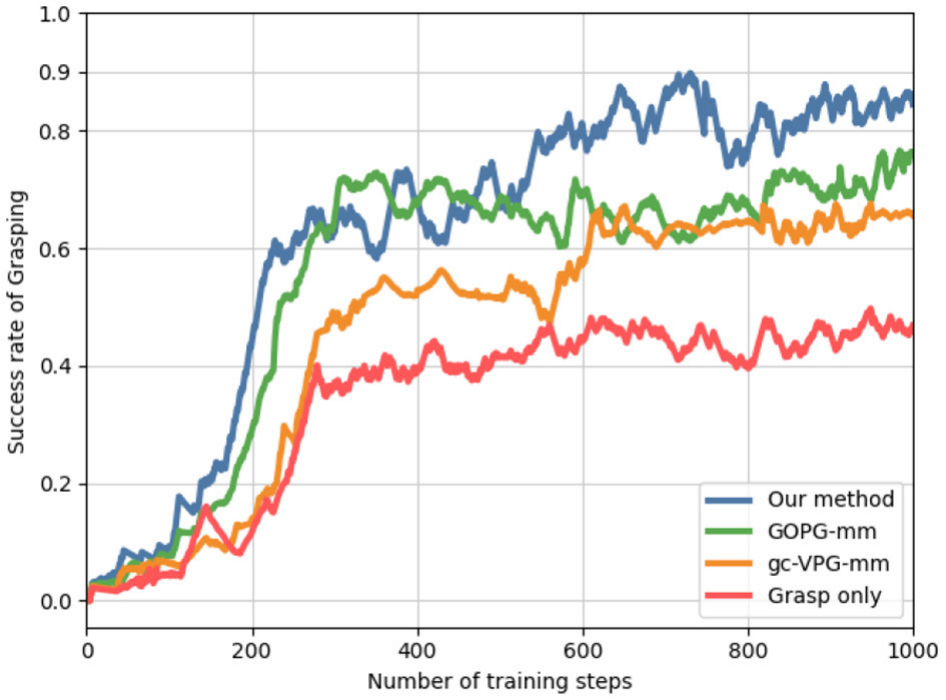
Multi-target grasping success rate reflecting the training performance of different methods.

Figure 6 shows the training performance of our method and others. Considering the multi-target task setting, we considered only grasp, gc-VPG-mm, and GOPG-mm as the comparison methods. Our approach achieved better performance over 1,000 training steps. Grasp was the only policy network obtained when we trained only the grasping net. The performances obtained by the other two methods also highlighted the necessity of pushing action for multi-target push-grasping in clutter.

#### 5.3.1. The test for multi-target

**Figure 7:**
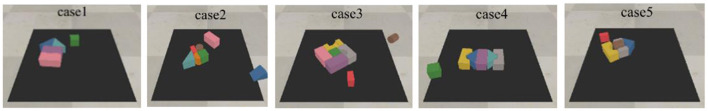
Five cases of challenging arrangements.

We conducted test experiments in random and challenging clutter scenarios. The random clutter scenes: A total of 20 objects randomly fell into the workspace, and the targets were three arbitrarily assigned objects among them. Notably, the training environment was the same, and the difference was that there were 10 falling objects and that three target objects were randomly assigned. For challenging scenarios, we used the following five arrangements: case 1, case 2, case 3, case 4, and case 5 (in Table, simplified to C1, C2, C3, C4, and C5), as shown in Figure 7, and the targets were three randomly assigned objects. These two categories of experiments were used to test the generalization ability of the learned policy and the effectiveness of our method.

We conducted 30 runs of each test, and the results are shown in Tables 1–4. The alphabet letter r corresponds to random arrangements and A corresponds to the average.

**Table 1:** The completion of multi-target experiments.

**Metric**	**Completion (%)**						
Arrangement	r	C1	C2	C3	C4	C5	A
Grasp only	50.8	58.6	69.8	61.4	54.3	51.2	57.7
gc-VPG	63.2	64.5	77.8	68.4	63.2	63.2	66.7
GOPG	55.6	70.6	87.5	88.0	82.4	77.8	77.0
Our System	**92.2**	**100**	**100**	**100**	81.2	**89.5**	**93.8**

**Table 2:** The grasp success of multi-target experiments.

**Metric**	**Grasp success (%)**						
Arrangement	r	C1	C2	C3	C4	C5	A
Grasp only	43.6	42.5	39.3	41.4	30.8	33.2	38.5
gc-VPG	53.4	48.0	44.4	48.7	38.3	39.6	45.4
GOPG	59.2	52.1	54.7	59.3	60.6	49.8	56.0
Our System	**73.7**	61.3	**89.1**	**81.9**	**67.2**	**78.4**	**75.3**

**Table 3:** The motion number of multi-target experiments.

**Metric**	**Motion number**						
Arrangement	r	C1	C2	C3	C4	C5	A
Grasp only	15.2	14.1	9.2	13.7	13.8	13.5	13.3
gc-VPG	10.5	12.7	8.9	7.9	8.3	11.0	9.9
GOPG	8.4	9.1	6.2	9.6	8.1	8.1	8.3
Our System	**7.2**	**8.4**	**3.9**	**5.1**	**7.6**	6.5	**6.5**

**Table 4:** The action efficiency of multi-target experiments.

**Metric**	**Action efficiency (%)**						
Arrangement	r	C1	C2	C3	C4	C5	A
Grasp only	31.5	23.8	33.2	22.8	24.7	24.2	26.7
gc-VPG	39.0	38.4	35.5	39.5	29.9	28.8	35.2
GOPG	36.3	27.4	49.1	33.4	39.7	43.9	38.3
Our System	**49.3**	**38.9**	**82.9**	**61.2**	**44.0**	48.1	**54.1**

The results showed that the performance of the proposed method was better than that of other methods. The main reasons were as follows: first, VPG and GOPG did not take multiple targets into consideration, and all targets were only captured one by one during the test, which affected the overall quality of task completion and decreased the value of various metrics. Second, the proposed method set a reward mechanism according to the graspable probability of each target so that the policy could gradually master the capability in the learning process to maximize the graspable probability with the fewest movements to improve the execution efficiency of the system and reduce the total number of actions.

In terms of task completion, although our system was slightly inferior to another method for the C4 scene, it was significantly superior to baseline methods in other experimental scenes. In terms of grasp success rate, our system also showed the best performance except for C1. In motion number and action efficiency, our system was also more dominant. In a comprehensive view of all metrics, the results of grasping alone were the lowest, suggesting that grasping with push-assisting can better complete tasks in clutter. The gc-VPG and GOPG were in the middle, among which GOPG was more advantageous. These two methods integrate push action, making the execution of grasping tasks in clutter more efficient. However, due to the lack of further consideration of multiple targets, the overall efficiency of the system was not as optimal.

#### 5.3.2. The ablation studies for multi-target

The experimental settings were consistent with those mentioned above, and the purpose of the experiment was to verify the performance of the modified VPG and GOPG in handling multi-target pushing and grasping.

In terms of task completion, the modified VPG and GOPG showed improved performance, with the GOPG-mm achieving the best results in C4. In terms of grasp success rate, the results of the ablation studies showed some improvements. Meanwhile, GOPG-mm earned the best score in C1. In terms of motion number and action efficiency, GOPG-mm obtained a noticeable improvement, and two of the metrics reached the optimal level. Therefore, the experiments indicated that gc-VPG-mm and GOPG-mm had some potential to complete multi-target push-grasping tasks. The results are recorded in Tables 5–8.

**Table 5:** The completion of multi-target experiments for ablation studies.

**Metric**	**Completion (%)**						
Arrangement	r	C1	C2	C3	C4	C5	A
gc-VPG-mm	65.3	68.4	93.8	74.2	81.2	86.4	78.2
GOPG-mm	68.7	77.8	92.6	93.8	**88.2**	84.2	84.2

**Table 6:** The grasp success of multi-target experiments for ablation studies.

**Metric**	**Grasp success (%)**						
Arrangement	r	C1	C2	C3	C4	C5	A
gc-VPG-mm	54.3	51.2	48.9	72.7	46.2	68.0	56.9
GOPG-mm	62.8	**63.1**	69.6	71.8	63.6	66.0	66.2

**Table 7:** The motion number of multi-target experiments for ablation studies.

**Metric**	**Motion number**						
Arrangement	r	C1	C2	C3	C4	C5	A
gc-VPG-mm	9.8	11.4	7.8	6.6	9.1	13.0	9.6
GOPG-mm	8.2	10.7	5.6	6.9	9.4	**5.8**	7.8

**Table 8:** The action efficiency of multi-target experiments for ablation studies.

**Metric**	**Action efficiency (%)**						
Arrangement	r	C1	C2	C3	C4	C5	A
gc-VPG-mm	41.8	40.2	39.8	47.6	39.2	30.1	39.8
GOPG-mm	42.3	43.6	54.0	44.6	42.5	**54.8**	47.0

#### 5.3.3. The single-target test

Under the condition that our system remained unchanged in the training process, we tested the single-target policy learned by the proposed method in clutter scenarios and compared it with GOPG. The test scenarios were divided into two types: random and challenging. The random one: 20 different objects fell into the workspace, one of which was the target. The challenging one: we used the same 5 cases as above, but the target was one randomly assigned object. We conducted 30 runs of each test, and the results are displayed in Tables 9–12.

**Table 9:** The completion of single-target experiments.

**Metric**	**Completion (%)**						
Arrangement	r	C1	C2	C3	C4	C5	A
GOPG	93.3	**93.3**	93.8	86.7	93.8	76.5	89.6
Our System	**100**	72.2	**100**	**93.8**	**100**	**87.5**	**92.3**

**Table 10:** The grasp success of single-target experiments.

**Metric**	**Grasp success (%)**						
Arrangement	r	C1	C2	C3	C4	C5	A
GOPG	55.5	**68.7**	66.9	64.1	29.4	77.9	60.4
Our System	**76.6**	41.3	**96.7**	**79.4**	**42.3**	**96.4**	**72.1**

**Table 11:** The motion number of single-target experiments.

**Metric**	**Motion number**						
Arrangement	r	C1	C2	C3	C4	C5	A
GOPG	5.1	**4.2**	3.6	**3.6**	4.3	5.2	4.3
Our System	**2.8**	4.7	**2.1**	3.6	**2.7**	**4.1**	**3.3**

**Table 12:** The action efficiency of single-target experiments.

**Metric**	**Action efficiency (%)**						
Arrangement	r	C1	C2	C3	C4	C5	A
GOPG	25.7	**26.6**	38.0	**30.9**	29.2	24.8	29.2
Our System	**52.9**	24.6	**48.9**	30.2	**42.3**	**26.6**	**37.6**

The experimental results showed that the performance of the proposed method was still well and above that of the compared method for a single target. Hence, our system was also competent for single-target push-grasping in clutter scenes. The proposed method referred to these single-target push-grasping methods and then improved and optimized them based on their ability to retain their ability to single-target push-grasp.

### 5.4. Real-world experiments

**Figure 8:**
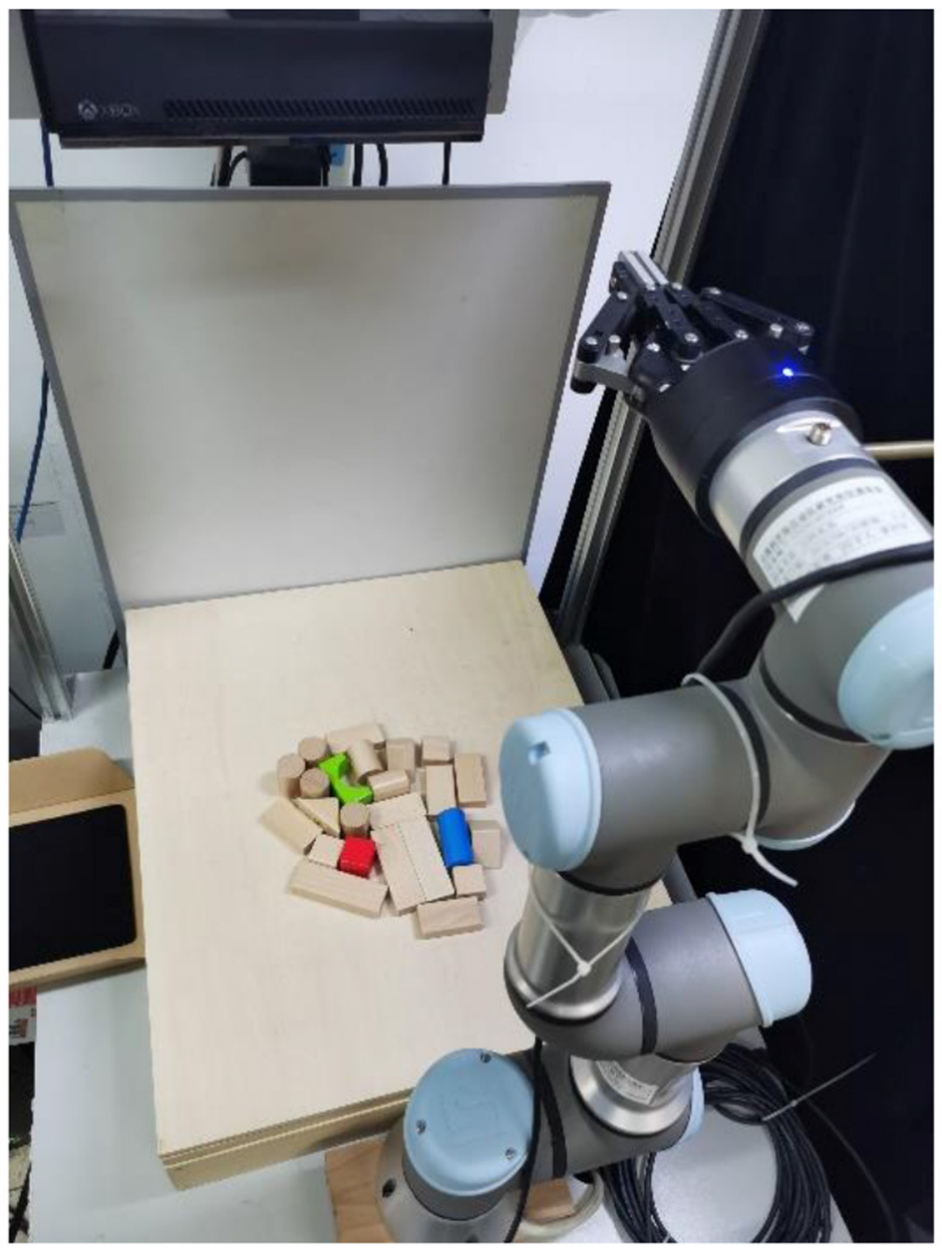
The real-world experiment system which comprises an UR3 robot arm equipped with a robotic-85 gripper and a Kinect2 camera mounted on top.

**Figure 9:**
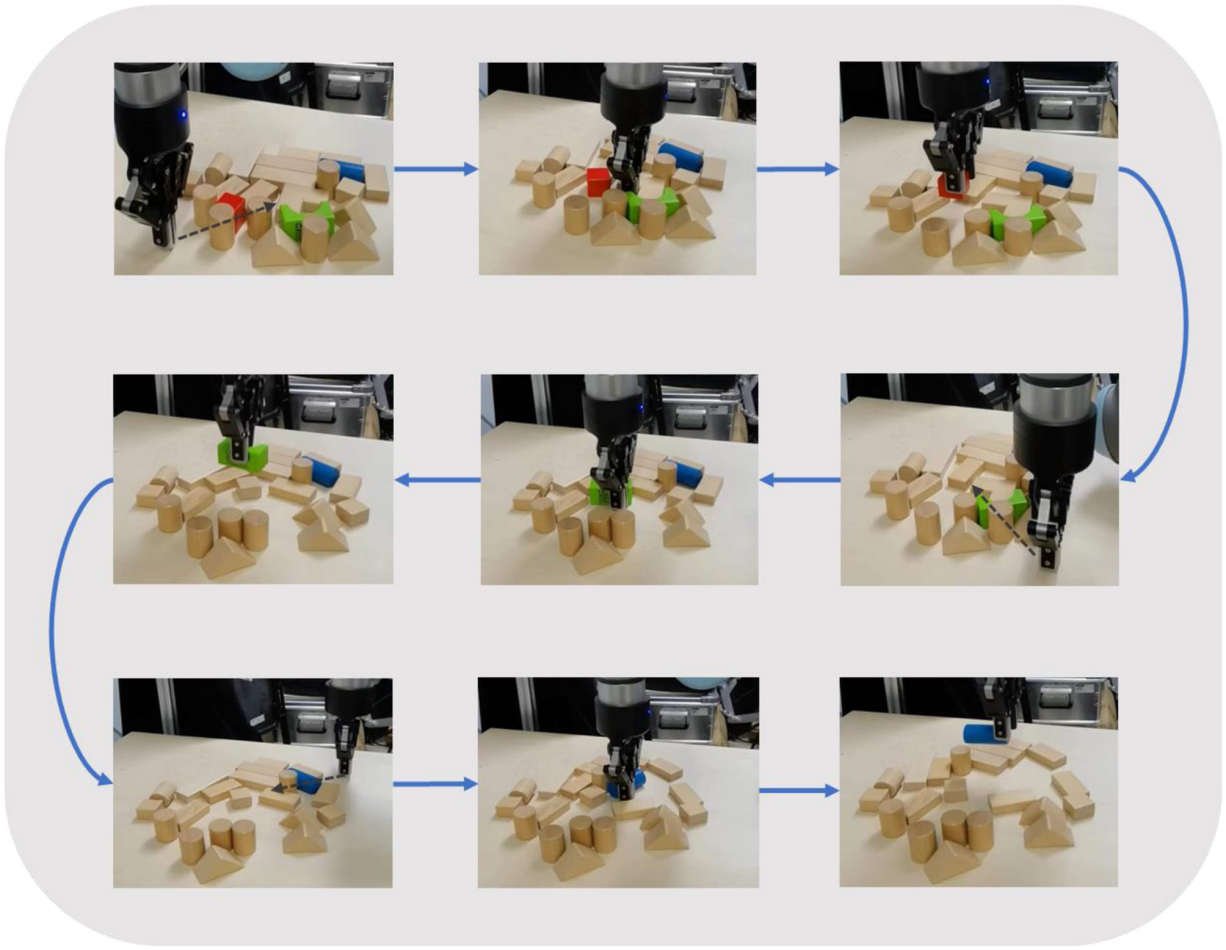
The multi-target push-grasping manipulations.

**Figure 10:**
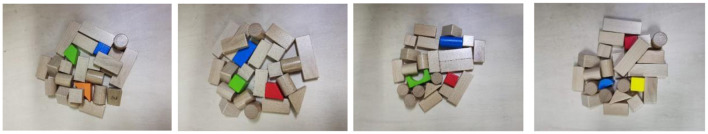
Four random clutter arrangements.

**Figure 11:**
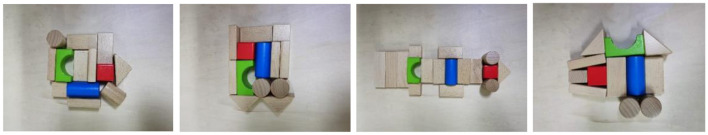
Four challenging arrangements.

In this section, we conducted real-world experiments to verify our system. Our existing system consisted of a UR3 robot arm with a ROBOTIQ-85 gripper, and the acquisition of the RGB-D image was performed using Kinect2 with 1920 × 1080 pixels (shown in Figure 8). Figure 9 shows the experimental process of the multi-target push-grasping manipulations of the whole system. The test cases included four random clutter arrangements in which the target objects were occluded (shown in Figure 10) and four setting-challenging arrangements (shown in Figure 11). The targets were colored objects, while the non-targets were wood-colored objects. The comparison method we used was GOPG-mm, which performed better than the other baselines.

In the real-world experiment, we executed 20 runs for each test. It should be noted that the policies of each method were obtained through training under simulation and transferred to the real system for direct application without retraining or fine-tuning. The average results for specific metrics such as completion rate, grasp success rate, motion number, and action efficiency are shown in Tables 13, 14 (where r, c, n, and A represent random, challenging, novel target arrangements, and average, respectively). Overall, our system was superior to the comparison method on all metrics. This also indicates that our policy can be effectively generalized to a real system.

**Table 13:** Completion and grasp success of real-world experiments.

**Metric**	**Completion (%)**				**Grasp success (%)**			
Arrangement	r	C	n	A	r	c	N	A
GOPG-mm	59.2	72.7	53.4	61.8	55.8	60.2	51.6	55.9
Our System	**86.4**	**91.3**	**80.6**	**86.1**	**68.4**	**73.5**	**62.7**	**68.2**

**Table 14:** Motion number and action efficiency of real-world experiments.

**Metric**	**Motion number**				**Action efficiency (%)**			
Arrangement	R	c	n	A	r	c	N	A
GOPG-mm	14.2	13.5	16.8	14.8	35.9	37.2	31.6	34.9
Our System	**10.3**	**9.2**	**13.1**	**10.9**	**43.2**	**48.1**	**39.8**	**43.7**

**Figure 12:**
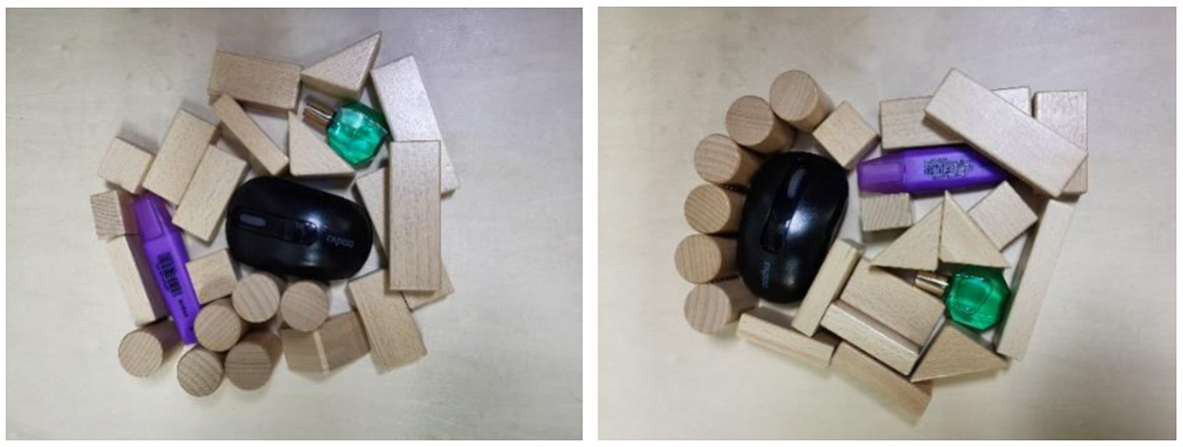
Two arrangements of novel target objects.

In addition, we tested the generalization ability of the system for novel target objects unseen during training. The targets were everyday objects with more complex shapes (shown in Figure 12). The results are shown in the tables above, indicating that our method can generalize to unseen objects and that the system performance is better than the baseline method. For the purple pen and eye drop, the system can accomplish the task well, while for the mouse, the success rate was relatively low. The more likely reason was that the shapes of the former are closer to certain training objects, but the latter lacked similar objects.

## 6. Conclusions

In this study, we studied the multi-target push-grasping task in clutter scenarios and conducted policy training under simulation based on model-free reinforcement learning. To incorporate the multi-target states and achieve the policy by gradually learning how to push and grasp efficiently multiple target objects, we fused the mask of each target, defined the graspable probability, and designed a reward mechanism. We evaluated the learned policy not only for simulation but also for the real world. The experiment results revealed that the proposed method achieves better results than other methods under random, challenging clutter scenarios and novel target objects in the real world.

Moreover, our method retained the effectiveness of single-target push-grasping, endowing our system with comprehensive capabilities and robust sim-to-real generalization.

In the future, we will study situations where the number of objects is larger and the shape of objects is more complex, further explore relevant scientific problems and propose feasible method.

## Data availability statement

The original contributions presented in the study are included in the article/supplementary material, further inquiries can be directed to the corresponding author.

## Author contributions

LW and YC: conceptualization and methodology. LW, YC, and ZLi: software. ZLiu: validation, resource gathering, supervision, project administration, and funding acquisition. LW: formal analysis, data curation, writing—original draft and review and editing, and visualization. LW and ZLi: investigation. All authors have read and approved the published version of the manuscript.

## References

[B1] Andrychowicz M. Wolski F. Ray A. Schneider J. Fong R. Welinder P.. (2017). “Hindsight experience replay,” in Advances in neural information processing systems. p. 30.

[B2] Bauza M. Rodriguez A. (2017). A probabilistic data-driven model for planar pushing 2017 IEEE International Conference on Robotics and Automation (ICRA). New York City: IEEE. 2017, 3008–3015. 10.1109/ICRA.2017.7989345

[B3] Bohg J. Morales A. Asfour T. Kragic D. (2013). Data-driven grasp synthesis—a survey. IEEE Transact. Robot. 30, 289–309. 10.1109/TRO.2013.2289018

[B4] Boularias A. Bagnell J. A. Stentz A. (2014). “Efficient optimization for autonomous robotic manipulation of natural objects,” in Proceedings of the AAAI Conference on Artificial Intelligence. Vol. 28 (AAAI Press). 10.1609/aaai.v28i1.9052

[B5] Boularias A. Bagnell J. A. Stentz A. (2015). “Learning to manipulate unknown objects in clutter by reinforcement Twenty-Ninth,” in AAAI Conference on Artificial Intelligence. 10.1609/aaai.v29i1.9378

[B6] Choi C. Schwarting W. DelPreto J. Rus D. (2018). Learning object grasping for soft robot hands. IEEE Robot. Automat. Lett. 3, 2370–2377. 10.1109/LRA.2018.2810544

[B7] Cosgun A. Hermans T. Emeli V. Stilman M. (2011). “Push planning for object placement on cluttered table surfaces,” in 2011 IEEE/RSJ International Conference on Intelligent Robots and Systems (IEEE). p. 4627–4632. 10.1109/IROS.2011.6094737

[B8] Danielczuk M. Mahler J. Correa C. Goldberg K. (2018). “Linear push policies to increase grasp access for robot bin picking,” in 2018 IEEE 14th International Conference on Automation Science and Engineering (CASE). New York City: IEEE. p. 1249–1256. 10.1109/COASE.2018.8560406

[B9] Deng J. Dong W. Socher R. Li L-J. Li K. Fei-Fei L. (2009). “Imagenet: A large-scale hierarchical image database,” in 2009 IEEE conference on computer vision and pattern recognition. New York City: IEEE. p. 248–255. 10.1109/CVPR.2009.520684826886976

[B10] Deng Y. Guo X. Wei Y. Lu K. Fang B. Guo D.. (2019). “Deep reinforcement learning for robotic pushing and picking in cluttered environment,” in 2019 IEEE/RSJ International Conference on Intelligent Robots and Systems (IROS). New York City: IEEE p. 619–626. 10.1109/IROS40897.2019.8967899

[B11] Dogar M. R. Srinivasa S. (2010). Push-grasping with dexterous hands: mechanics and a method 2010 IEEE/RSJ International Conference on Intelligent Robots and Systems. New York City: IEEE. 2010, 2123–2130. 10.1109/IROS.2010.5652970

[B12] Fang H.-S. Wang C. Gou M. Lu C. (2020). “Graspnet-1billion: A large-scale benchmark for general object grasping,” in Proceedings of the IEEE/CVF Conference on Computer Vision and Pattern Recognition (IEEE), 11444–11453. 10.1109/CVPR42600.2020.01146

[B13] Hang K. Morgan A. S. Dollar A. M. (2019). Pre-grasp sliding manipulation of thin objects using soft, compliant, or underactuated hands. IEEE Robot. Automation Lett. 4, 662–669. 10.1109/LRA.2019.2892591

[B14] Huang B. Han S. D. Boularias A. Yu J. (2021a). “Dipn: Deep interaction prediction network with application to clutter removal,” in 2021 IEEE International Conference on Robotics and Automation (ICRA). New York City: IEEE. p. 4694–4701. 10.1109/ICRA48506.2021.9561073

[B15] Huang B. Han S. D. Yu J. Boularias A. (2021b). Visual foresight trees for object retrieval from clutter with non-prehensile rearrangement. IEEE Robot. Automat. Lett. 7, 231–238. 10.1109/LRA.2021.3123373

[B16] Huang G. Liu Z. Van Der Maaten L. Weinberger K. Q. (2017). “Densely connected convolutional networks,” in Proceedings of the IEEE Conference on Computer Vision and Pattern Recognition (IEEE), 4700–4708. 10.1109/CVPR.2017.243

[B17] Ioffe S. Szegedy C. (2015). “Batch normalization: Accelerating deep network training by reducing internal covariate shift,” in International conference on machine learning. New York City: PMLR. p. 448–456.35496726

[B18] Kalashnikov D. Irpan A. Pastor P. Ibarz J. Herzog A. Jang E.. (2018). “Scalable deep reinforcement learning for vision-based robotic manipulation,” in Conference on Robot Learning. New York City: PMLR p. 651–673.

[B19] Kiatos M. Malassiotis S. (2019). “Robust object grasping in clutter via singulation” in 2019 International Conference on Robotics and Automation (ICRA). p. 1596–1600. 10.1109/ICRA.2019.8793972

[B20] Kurenkov A. Taglic J. Kulkarni R. Dominguez-Kuhne M. Garg A. Martin-Martin R.. (2020). “Visuomotor mechanical search: Learning to retrieve target objects in clutter,” in 2020 IEEE/RSJ International Conference on Intelligent Robots and Systems (IROS). New York City: IEEE. p. 8408–8414. 10.1109/IROS45743.2020.9341545

[B21] Lu Q. Van der Merwe M. Sundaralingam B. Hermans T. (2020). Multifingered grasp planning via inference in deep neural networks: outperforming sampling by learning differentiable models. IEEE Robot. Automat. Lett. 27, 55–65. 10.1109/MRA.2020.2976322

[B22] Mahler J. Goldberg K. (2017). “Learning deep policies for robot bin picking by simulating robust grasping sequences,” in Conference on robot learning. New York City: PMLR. p. 515–524.

[B23] Mahler J. Liang J. Niyaz S. Laskey M. Doan R. Liu X.. (2017). Dex-net 2.0: Deep learning to plan robust grasps with synthetic point clouds and analytic grasp metrics. arXiv [Preprint]. arXiv: 1703.09312. Available online at: https://arxiv.org/pdf/1703.09312.pdf

[B24] Moll M. Kavraki L. Rosell J. (2017). Randomized physics-based motion planning for grasping in cluttered and uncertain environments. IEEE Robot. Automat. Lett. 3, 712–719. 10.1109/LRA.2017.2783445

[B25] Nair V. Hinton G. E. (2010). “Rectified linear units improve restricted boltzmann machines,” in Proceedings of the 27th International Conference on Machine Learning (ICML-10). p. 807–814.

[B26] Ovur S. E. Zhou X. Qi W. Zhang L. Hu Y. Su H. (2021). A novel autonomous learning framework to enhance sEMG-based hand gesture recognition using depth information. Biomed. Signal Proc. Control. 66, 102444. 10.1016/j.bspc.2021.102444

[B27] Qi W. Liu X. Zhang L. Wu L. Zang W. Su H. (2021). Adaptive sensor fusion labeling framework for hand pose recognition in robot teleoperation. Assem. Autom. 10.1108/AA-11-2020-0178

[B28] Qi W. Wang N. Su H. Aliverti A. (2022). DCNN based human activity recognition framework with depth vision guiding. Neurocomputing, 486, 261–271. 10.1016/j.neucom.2021.11.044

[B29] Rodriguez A. Mason M. T. Ferry S. (2012). From caging to grasping. Int. J. Robotics Res. 31, 886–900. 10.1177/0278364912442972

[B30] Sarantopoulos I. Kiatos M. Doulgeri Z. Malassiotis S. (2020). “Split deep q-learning for robust object singulation,” in 2020 IEEE International Conference on Robotics and Automation (ICRA). New York City: IEEE. p. 6225–6231. 10.1109/ICRA40945.2020.9196647

[B31] Song C. Boularias A. (2020). Learning to slide unknown objects with differentiable physics simulations. arXiv [Preprint]. arXiv: 2005.05456. Available online at: https://arxiv.org/pdf/2005.05456.pdf

[B32] Ten Pas A. Platt R. (2018). “Using geometry to detect grasp poses in 3d point clouds,” in Robotics Research. Cham: Springer. 2018:307–324. 10.1007/978-3-319-51532-8_19

[B33] Xu K. Yu H. Lai Q. Wang Y. Xiong R. (2021). Efficient learning of goal-oriented push-grasping synergy in clutter. IEEE Robot. Automat. Lett. 6, 6337–6344. 10.1109/LRA.2021.3092640

[B34] Yang Y. Liang H. Choi C. (2020). A deep learning approach to grasping the invisible. IEEE Robot. Automat. Lett. 5, 2232–2239. 10.1109/LRA.2020.2970622

[B35] Zeng A. Song S. Welker S. Lee J. Rodriguez A. Funkhouser T. (2018). Learning synergies between pushing and grasping with self-supervised deep reinforcement learning[C]//2018 IEEE/RSJ International Conference on Intelligent Robots and Systems (IROS). New York City: IEEE. 2018, 4238–4245. 10.1109/IROS.2018.8593986

[B36] Zhang Y. Xie L. Li Y. Li Y. (2023). A neural learning approach for simultaneous object detection and grasp detection in cluttered scenes. Front. Comput. Neurosci. 17, 1110889. 10.3389/fncom.2023.1110889 36890968 PMC9986287

